# Plasmid-resolved genomics and machine learning-assisted profiling reveal *Enterobacter soli* as an overlooked host of a *bla*_NDM-1_-bearing IncX3 plasmid

**DOI:** 10.3389/fmicb.2026.1909491

**Published:** 2026-08-04

**Authors:** Yao Li, Chang Peng, Liqun Wu, Juan Wang, Meiling Li, Xiaoli Liu, Xiaomin Wu, Lin Gong

**Affiliations:** 1 Wuhan Center for Disease Control and Prevention, Wuhan, Hubei, China; 2 People's Hospital of Huangpi District, Wuhan, Hubei, China; 3 State Key Laboratory of Environment Health (Incubation), Key Laboratory of Environment and Health (Ministry of Education), Key Laboratory of Health Effects of Environmental Pollution (Ministry of Ecology and Environment), School of Public Health, Tongji Medical College, Huazhong University of Science and Technology, Wuhan, Hubei, China

**Keywords:** *bla*_NDM-1_, carbapenem resistance, *Enterobacter soli*, IncX3 plasmid, machine-learning-assisted profiling, plasmid-resolved genomics

## Abstract

**Background:**

Plasmid-mediated carbapenem resistance is a major public health concern, yet the role of under-characterized *Enterobacter* species in carrying clinically important resistance plasmids remains insufficiently defined. In particular, plasmid-resolved genomic information on *bla*_NDM-1_-positive *Enterobacter soli* is scarce, and it remains unclear whether clinical *E. soli* can harbor conserved *bla*_NDM-1_-bearing IncX3 plasmids related to those circulating among other Enterobacterales hosts.

**Methods:**

In this study, we investigated carbapenem-resistant *E. soli* strain HP81 using complete genome sequencing, antimicrobial susceptibility testing, resistome analysis, plasmid characterization, comparative genomics, and machine learning-assisted plasmid profiling. A public dataset of 1,064 IncX3 plasmids was further used to evaluate whether the key plasmid of strain HP81 conformed to a broader *bla*_NDM_-positive IncX3 plasmid profile.

**Results:**

Strain HP81 was identified as a multidrug-resistant *E. soli* isolate belonging to sequence type 3367 (ST3367) and carried four plasmids. The key plasmid, pHP81-3, was a 54,652-base-pair IncX3 plasmid carrying *bla*_NDM-1_, *ble*, *bla*_SHV-12_, multiple mobile genetic elements, and transfer-associated genes. Comparative analysis showed that pHP81-3 was closely related to *bla*_NDM_-bearing IncX3 plasmids from other Enterobacterales hosts and contained a conserved *bla*_NDM-1_–*ble–trpF–dsbD–cutA* resistance module with variable flanking regions. Machine-learning-assisted profiling assigned pHP81-3 to a *bla*_NDM_-positive IncX3 plasmid profile, and a without-*ble* sensitivity model indicated that this classification was not solely dependent on the *bla*_NDM_-associated *ble* feature.

**Conclusion:**

These findings provide plasmid-resolved evidence that clinical *E. soli* can serve as an overlooked host of a conserved IncX3-*bla*_NDM_ resistance plasmid. By combining complete plasmid characterization, comparative genomics, and machine learning-assisted profiling of public IncX3 plasmids, this study expands current understanding of the host range and genomic context of IncX3-*bla*_NDM_ plasmids and provides a quantitative framework for profiling clinically important *bla*_NDM_-positive IncX3 plasmid signatures.

## 1 Introduction

Carbapenem resistance is a major global public health threat because it severely limits treatment options for infections caused by multidrug-resistant Gram-negative bacteria. Among carbapenemase genes, *bla*_NDM-1_ is particularly concerning because it is frequently carried by transferable plasmids and can disseminate across diverse Enterobacterales hosts. Horizontal plasmid transfer plays a central role in the spread of *bla*_NDM-1_ and the evolution of carbapenem resistance (Dong et al., 2022). Carbapenemase genes are often embedded in complex mobile genetic backgrounds rather than occurring as isolated resistance determinants. Previous studies have reported *bla*_NDM_, *bla*_IMP_, and other resistance genes on hybrid plasmids, megaplasmids, and mosaic multidrug-resistance regions (Jia et al., 2022; Zhong et al., 2022; Li et al., 2023). Carbapenemase genes can also move within and between bacterial species and persist through multispecies transmission in clinical settings, including in less commonly reported species (Zhang et al., 2024; Liu et al., 2025a; Shin et al., 2025). These findings indicate that plasmid backbones, plasmid diversity, and mobile genetic elements are key drivers of carbapenemase dissemination, making closed-plasmid analysis important for tracing resistance modules (Koh et al., 2025; Lerminiaux et al., 2025; Mauffrey et al., 2025; Mitra et al., 2025; Parker et al., 2025).

Enterobacter spp. are increasingly recognized as hosts of carbapenemase genes in clinical and non-clinical settings. Carbapenemase-producing Enterobacter isolates have been recovered from environmental, food-associated, and clinical sources (Börjesson et al., 2022; Che et al., 2023; Spahr et al., 2025; Teixeira et al., 2025; Zhang et al., 2025), and clinical studies have identified carbapenemase-producing *E. asburiae* and members of the *E. cloacae* complex, including high-risk sequence types, bloodstream infection isolates, and strains carrying newly described carbapenemase variants (Mattioni Marchetti et al., 2024; Kawase et al., 2025; Wang et al., 2026). More broadly, recent studies have highlighted the diversity and dissemination of plasmid-associated antimicrobial resistance genes across different ecological niches (Munir et al., 2025), biofilm formation among pathogenic Enterobacterales isolated from infected rabbit gastrointestinal tracts (Lenchenko et al., 2024), and the application of *Enterobacter cloacae* in agricultural environments as a plant-associated biofertilizer (Ruiz et al., 2025), further illustrating the ecological diversity of Enterobacterales beyond human clinical settings. Regional surveillance and recent genomic epidemiology studies have further suggested that carbapenemase-producing Enterobacter isolates may be geographically widespread and overlooked in routine surveillance (Emeraud et al., 2024; Cirkovic and Brkic, 2025; Teixeira et al., 2025). The recent identification of *bla*_FRI-12_ on an IncR plasmid in *E. asburiae* also highlights the capacity of this genus to acquire emerging plasmid-borne carbapenemase genes (Mataseje et al., 2024). *Enterobacter soli* was originally described as a lignin-degrading bacterium isolated from soil (Manter et al., 2011), whereas clinical reports of this species remain scarce. Its historical association with environmental niches and limited representation in clinical genomic studies may have contributed to its underrecognition relative to better-characterized *Enterobacter* species. Consequently, its resistance background, plasmid architecture, and epidemiological relevance remain poorly characterized. A previous report described a clinical *E. soli* strain, 140,044, isolated from human blood and carrying the non-NDM carbapenemase gene *bla*_IMP-4_ (Feng and Zong, 2024). Nevertheless, reports of carbapenemase-producing *E. soli* remain scarce, and the occurrence and complete plasmid context of *bla*_NDM-1_ in this species remain insufficiently characterized. In particular, it remains unclear whether clinical *E. soli* can carry a *bla*_NDM-1_-bearing IncX3 plasmid with a conserved resistance module related to those circulating among other Enterobacterales hosts.

The spread of carbapenem resistance depends not only on resistance genes, but also on the plasmid backbones and mobile elements that carry them. Conjugative plasmids, multidrug-resistance plasmid backbones, integrons, and insertion sequences provide genetic contexts in which carbapenemase genes can be captured, maintained, and transferred. Comparative studies of IncX and other transferable plasmids have shown that successful resistance plasmids may combine efficient conjugation with relatively limited fitness costs (Yang et al., 2025), while large multidrug-resistance plasmids can bring *bla*_NDM-1_ together with additional *β*-lactamase genes (Liu et al., 2025b). Insertion sequences, especially *IS26*-mediated recombination, have been linked to the evolution and spread of *bla*_NDM_-carrying plasmids (Zhao et al., 2021; Acman et al., 2022; Tavares et al., 2025), and recent studies of carbapenem-resistant Enterobacterales have further highlighted the contribution of diverse mobile genetic elements to carbapenemase dissemination in clinical settings (Mitra et al., 2025). Mobile elements may also bring resistance and virulence-associated traits into the same transferable structure, potentially increasing the survival and spread of high-risk pathogens (Han et al., 2025). Similar plasmid-based studies have shown that resistance genes may be embedded in insertion-sequence-flanked composite structures, supporting the role of mobile elements in plasmid-borne resistance-region evolution (Ermakova et al., 2020).

IncX3 plasmids have attracted particular attention because they are frequently associated with the dissemination of clinically important carbapenemase genes among Enterobacterales. Recent studies have shown that IncX3 plasmids can carry *bla*_NDM_, *bla*_KPC_, and *bla*_OXA-181_ and can spread across different countries and bacterial species. An in silico analysis of *bla*_NDM_-harboring plasmids in *Klebsiella pneumoniae* identified single-replicon IncX3 plasmids as important carriers of *bla*_NDM_, with most ranging from approximately 46 to 57 kb in size (Zeng et al., 2022); comparative genomic analyses have also demonstrated the highly conserved backbone organization of IncX3 plasmids (Liakopoulos et al., 2018). Recent Frontiers in Microbiology studies have also reported *bla*_NDM_-bearing plasmids and complex multidrug-resistance plasmid backgrounds in clinical Enterobacterales, including *Enterobacter hormaechei* and *Citrobacter freundii*, highlighting the continued emergence of carbapenemase-encoding plasmids in diverse bacterial hosts (Guo et al., 2022; Yang et al., 2022; Zeng et al., 2022; Yuan et al., 2023; Liu et al., 2024).

Despite increasing reports of carbapenem-resistant *Enterobacter* spp., the contribution of *Enterobacter soli* to plasmid-mediated carbapenem resistance remains insufficiently understood. Three specific knowledge gaps are evident. First, clinical genome-resolved data on carbapenem-resistant *E. soli* remain scarce, limiting understanding of its resistance background and plasmid content. Second, the complete plasmid architecture and mobile-element context of *bla*_NDM-1_ in *E. soli* are unclear, particularly whether this species can harbor an IncX3-*bla*_NDM_ plasmid similar to those circulating among other Enterobacterales hosts. Third, although IncX3 plasmids have been widely compared by comparative genomics, quantitative profiling of *bla*_NDM_-positive IncX3 plasmid signatures using broad public plasmid datasets remains limited.

To address these gaps, we performed plasmid-resolved genomic analysis of clinical *E. soli* strain HP81 by integrating phylogenomic placement, resistome profiling, complete plasmid characterization, comparative plasmid analysis, and machine-learning-assisted profiling of public IncX3 plasmids. The originality of this study lies in three aspects: it provides plasmid-resolved evidence that clinical *E. soli* can act as an overlooked host of a *bla*_NDM-1_-bearing IncX3 plasmid; it resolves the backbone structure, resistance region, mobile-element context, and cross-host relatedness of the key plasmid pHP81-3; and it introduces a reproducible machine-learning-assisted framework to quantitatively assess whether pHP81-3 conforms to a conserved *bla*_NDM_-positive IncX3 plasmid profile. Together, these findings expand current knowledge of the host range and genomic context of IncX3-*bla*_NDM_ resistance plasmids and provide a quantitative perspective for profiling clinically important resistance plasmid signatures.

## 2 Materials and methods

### 2.1 Bacterial isolate and clinical background

Strain HP81 was recovered from a blood culture obtained from a 61-year-old male patient admitted to a hospital in Wuhan, China, in November 2019. The blood culture was obtained during routine clinical diagnostic evaluation rather than through an active surveillance or colonization-screening program. The patient presented with gastrointestinal hemorrhage and was further diagnosed with a duodenal mass, infection, hepatic insufficiency, and schistosomiasis-associated liver disease. The available retrospective records contained no documented history of previous *E. soli* infection in this patient and did not establish *E. soli* as the causative pathogen of a primary bloodstream infection. Following its isolation in November 2019, strain HP81 was cryopreserved at −80 °C. In 2025, it was retrieved from storage and selected for antimicrobial susceptibility testing, complete genome sequencing, and retrospective genomic characterization as part of an ongoing surveillance program for carbapenem-resistant Enterobacterales in Wuhan.

### 2.2 Antimicrobial susceptibility testing and preliminary identification

Antimicrobial susceptibility testing and biochemical identification were performed using the VITEK 2 Compact system (bioMérieux, Marcy-l’Étoile, France). Minimum inhibitory concentration (MIC) values were interpreted according to the Clinical and Laboratory Standards Institute (CLSI) M100 guidelines, 36th edition, 2026. The resulting antimicrobial susceptibility profile was used to define the carbapenem-resistant multidrug-resistant phenotype of strain HP81. Initial routine identification was further re-evaluated by genome-based analyses after whole-genome sequencing.

### 2.3 Genomic DNA extraction, library construction, and whole-genome sequencing

Genomic DNA was extracted using the SDS method (Lim et al., 2016). DNA quality and concentration were assessed by agarose gel electrophoresis and Qubit fluorometric quantification. For long-read sequencing, a > 10-kb insert library was constructed using the 1D ligation sequencing kit for the PromethION platform. For short-read sequencing, a paired-end library with an approximately 350-bp insert size was prepared using the NEBNext Ultra DNA Library Prep Kit for Illumina (NEB, USA). Whole-genome sequencing was performed on the PromethION platform and the Illumina NovaSeq PE150 platform at Beijing Novogene Bioinformatics Technology Co., Ltd.

### 2.4 Genome assembly, quality assessment, and strain re-identification

Raw Illumina reads were quality filtered using fastp (Chen et al., 2018) and initially assembled using SPAdes (Bankevich et al., 2012). Hybrid assembly was subsequently performed using both filtered Illumina short reads and long reads with Unicycler (Wick et al., 2017) to obtain the complete genome sequence. Assembly quality was evaluated using QUAST (Gurevich et al., 2013) and BUSCO (Manni et al., 2021). The final complete assembly was separated into chromosomal and plasmid sequences for downstream analyses. Species identification was confirmed by average nucleotide identity (ANI) analysis against reference/type genomes. The isolate showed an ANI value of 99.03% with the *Enterobacter soli* reference/type genome, supporting its assignment to *E. soli*. Sequence type assignment was conducted using the MLST service of the Center for Genomic Epidemiology (Larsen et al., 2012).

### 2.5 Genome annotation and identification of resistance genes, plasmid replicons, and insertion sequences

Bakta (Schwengers et al., 2021) was used as the primary annotation source for downstream genome and plasmid analyses and for the gene labels shown in the figures. Prokka (Seemann, 2014) and RAST (Aziz et al., 2008) were used as complementary annotation tools, whereas BLAST searches against the NCBI RefSeq database (Altschul et al., 1990) were used for manual verification of uncertain or inconsistent annotations. When discrepancies occurred, the corresponding loci were manually reviewed, and the best-supported annotations, based on sequence identity, query coverage, and consistency with the local genomic context, were retained. Acquired antimicrobial resistance genes were identified using ResFinder (Bortolaia et al., 2020), and plasmid replicons were detected using PlasmidFinder (Carattoli et al., 2014). Putative insertion sequences surrounding resistance loci were identified using ISfinder (Siguier, 2006). The genome record deposited in NCBI was subsequently annotated using version 6.11 of the NCBI Prokaryotic Genome Annotation Pipeline (Tatusova et al., 2016).

### 2.6 Comparative phylogenomic analysis of *Enterobacter soli*

**Figure 1:**
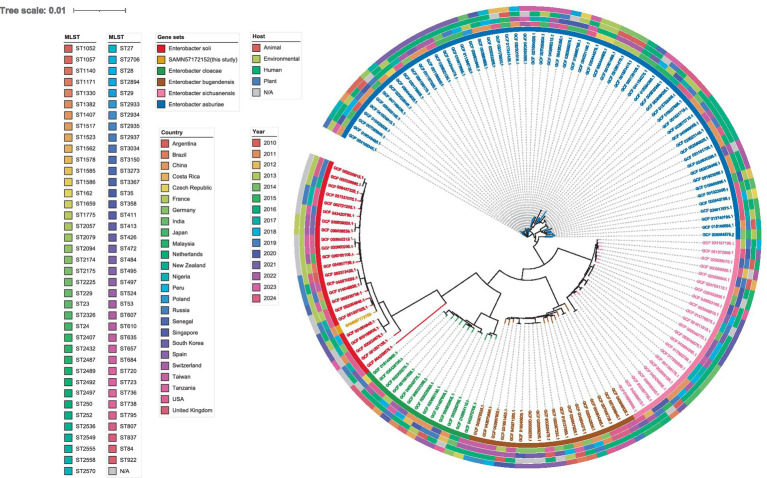
Core-genome phylogenetic placement of *Enterobacter soli* strain HP81 among publicly available Enterobacter genomes. Strain HP81 clustered within the *E. soli* lineage and represented ST3367. No external outgroup was included, and the tree was displayed as an unrooted phylogeny.

**Figure 2:**
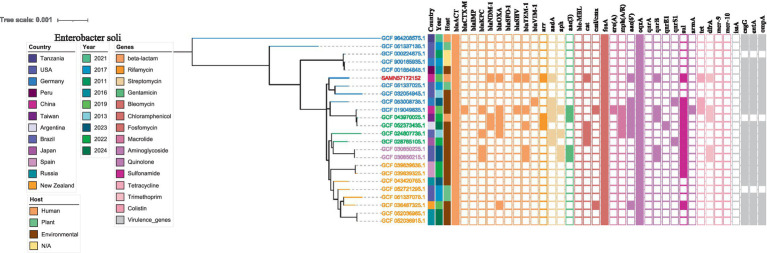
Comparative heatmap of epidemiological and genomic features among *Enterobacter soli* isolates. The heatmap shows the country, year, source, antimicrobial resistance genes, and representative virulence-associated genes of the analyzed genomes, with strain HP81 highlighted.

To determine the phylogenetic position of strain HP81 within the *Enterobacter soli* population, publicly available *Enterobacter* genomes, including available *E. soli* genomes, were downloaded from the NCBI database and analyzed together with the study isolate. Genomes with low assembly quality, highly fragmented assemblies, or ambiguous species assignment were excluded from the comparative dataset. A core-gene phylogenomic tree was constructed using GToTree (Lee, 2019) from strain HP81 and 136 publicly available *Enterobacter* genomes. No external outgroup was included, and the resulting tree was analyzed and displayed as an unrooted phylogeny in iTOL (Letunic and Bork, 2021). Metadata including sequence type, country, year, host source, and representative gene features were integrated into the phylogenetic display. The resulting phylogenetic framework and associated metadata matrix are shown in Figures 1, 2.

### 2.7 Plasmidome analysis and circular plasmid visualization

**Figure 3:**
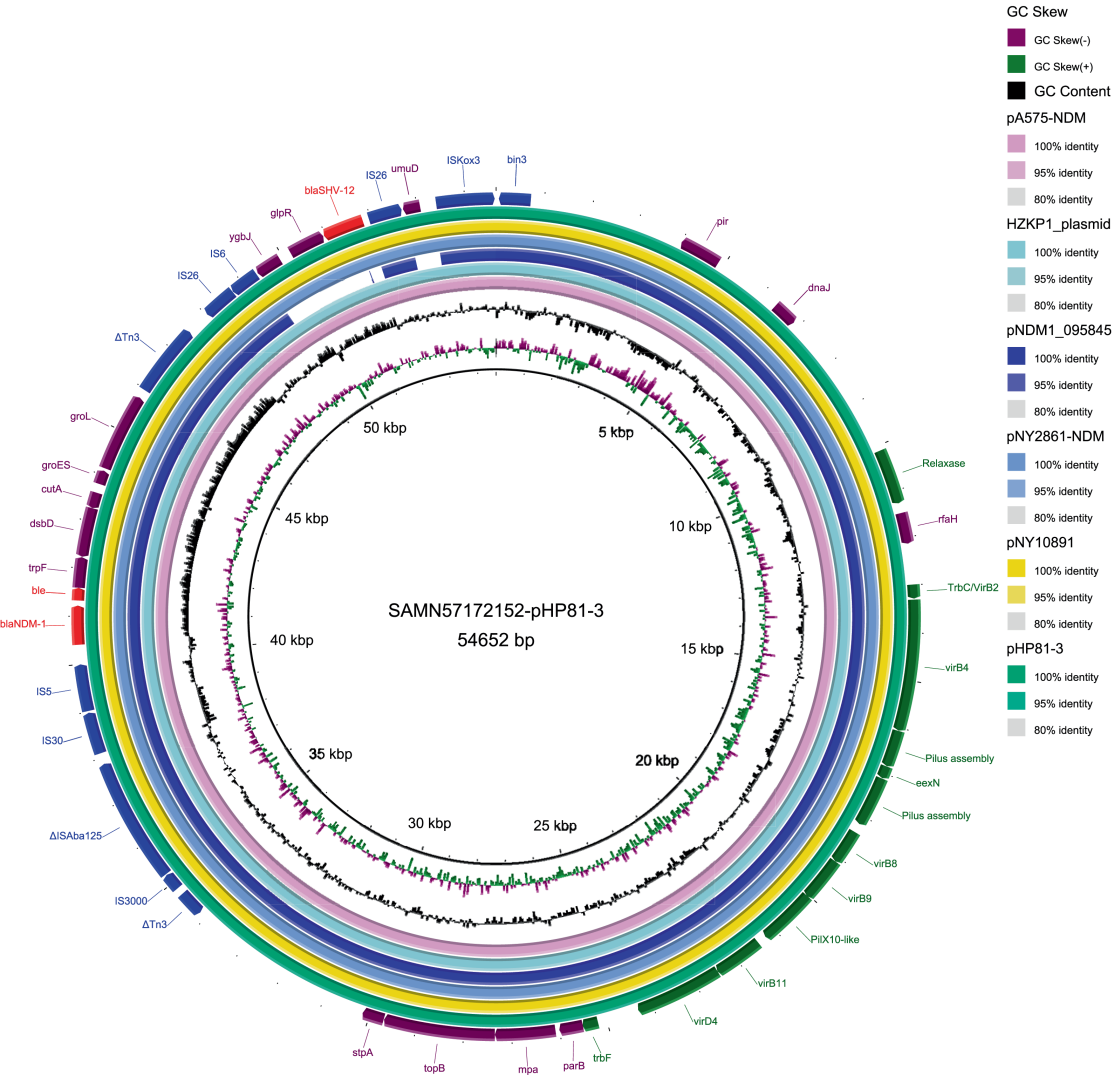
Circular map of pHP81-3. Colored rings indicate sequence similarity to related plasmids, and inner tracks show GC content and GC skew. Key backbone and accessory regions are annotated.

Complete plasmid sequences were extracted from the final genome assembly and analyzed individually. Circular plasmid maps were generated using BRIG (Alikhan et al., 2011) to visualize plasmid size, backbone organization, coding sequence distribution, and accessory genetic content. The circular map of pHP81-3 is shown in Figure 3, whereas the circular maps of the remaining three plasmids are provided in Supplementary Figure S1.

### 2.8 Comparative analysis of the *bla*_NDM-1_-carrying plasmid

To investigate the relationship of the *bla*_NDM-1_-carrying plasmid pHP81-3 to related plasmids, homologous plasmid sequences were retrieved from public databases using BLAST searches (Altschul et al., 1990). Comparative plasmid visualization was performed using BRIG (Alikhan et al., 2011). A whole-plasmid distance-based tree was generated with Mashtree (Katz et al., 2019) and visualized in iTOL (Letunic and Bork, 2021) to compare pHP81-3 with representative *bla*_NDM_-bearing plasmids from related Enterobacter spp. and other Enterobacterales hosts.

### 2.9 Analysis of the genetic environment of the *bla*_NDM-1_-associated resistance region

**Figure 4:**
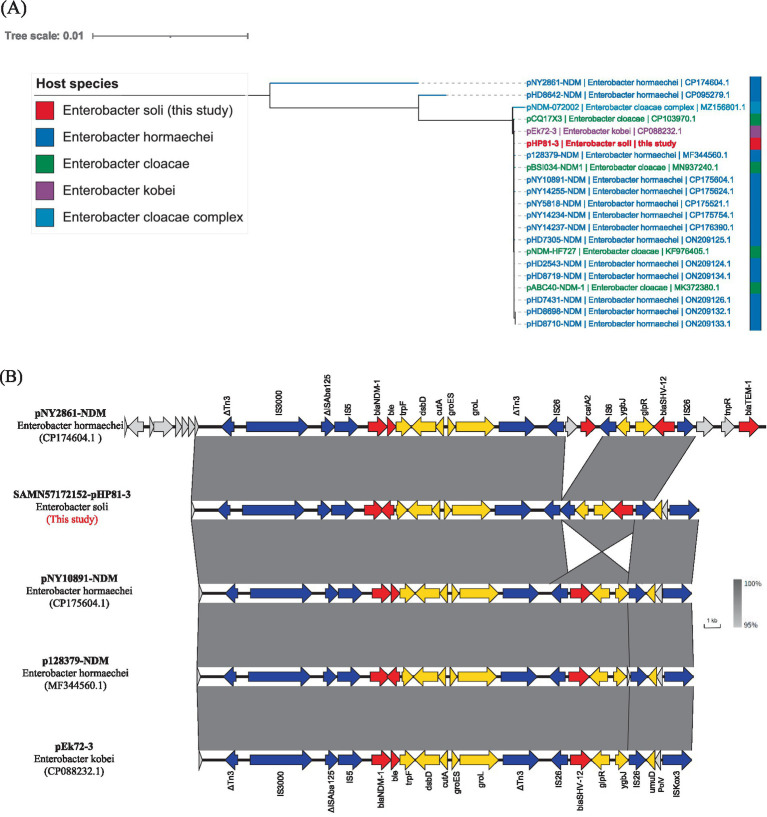
Phylogenetic and comparative genomic analysis of pHP81-3 and related *bla*_NDM_-bearing plasmids. **(A)** Whole-plasmid distance-based tree generated using Mashtree. **(B)** Comparative alignment of *bla*_NDM-1_-associated resistance regions, highlighting the conserved core module and variable flanking regions.

**Figure 5:**
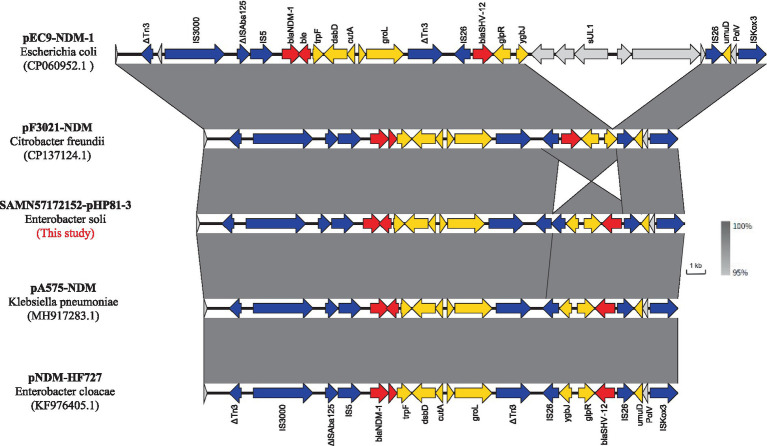
Cross-species comparison of *bla*_NDM-1_-associated resistance regions in Enterobacterales plasmids. Plasmids from *Enterobacter soli* HP81, *Klebsiella pneumoniae*, *Enterobacter cloacae*, *Citrobacter freundii*, and *Escherichia coli* were compared. Grey shading indicates nucleotide identity. The *bla*_NDM-1_–*ble–trpF–dsbD–cutA* region was conserved, whereas the flanking regions showed local deletions, inversions, and rearrangements. Comparison plasmids were selected from high-similarity BLASTn hits to represent diverse Enterobacterales host backgrounds.

To examine the local genomic context of *bla*_NDM-1_, the target gene and its flanking regions were extracted from pHP81-3. Homologous regions in related plasmids were identified using BLASTn (Altschul et al., 1990). Pairwise BLASTn comparisons were performed using the default BLAST+ parameters. For visualization in Easyfig, alignments with an E-value of ≤1 × 10^−3^ and nucleotide identity of ≥95% were displayed, with no additional minimum alignment-length threshold applied. Comparative synteny maps were generated using Easyfig (Sullivan et al., 2011). Conserved genes, antimicrobial resistance genes, and mobile genetic elements were manually curated to evaluate conservation and local structural variation of the *bla*_NDM-1_-associated region. The same parameters were applied to the resistance-region alignments shown in Figures 4B and 5. For Figure 5, candidate plasmids were first identified based on high nucleotide identity and query coverage to the *bla*_NDM-1_-associated region of pHP81-3, after which representative plasmids from different Enterobacterales hosts were selected to capture host diversity.

### 2.10 Machine-learning-assisted profiling of *bla*_NDM_-positive IncX3 plasmids

To evaluate whether pHP81-3 conforms to broader *bla*_NDM_-positive IncX3 plasmid profiles, a public IncX3 plasmid dataset was compiled for random forest-based classification. A total of 1,064 IncX3 plasmids were included, comprising 577 *bla*_NDM_-positive and 487 non-*bla*_NDM_ plasmids. Eleven plasmid-associated features were extracted: plasmid length, GC content, coding sequence (CDS) count, antimicrobial resistance (AMR) gene count, *ble* presence, *bla*_SHV_ presence, *IS3000* presence, *IS5* presence, *IS26* presence, *Tn3* presence, and relaxase presence. A random forest classifier with 1,000 decision trees, balanced class weights, and a fixed random seed was implemented using scikit-learn. Model performance was assessed using an independent test set, receiver operating characteristic analysis, area under the curve, and five-fold stratified cross-validation. Feature contributions were interpreted using random forest feature importance and SHapley Additive exPlanations (SHAP). A without-*ble* sensitivity model was additionally constructed to examine whether classification remained robust after excluding the *bla*_NDM_-associated *ble* feature.

## 3 Results

### 3.1 Antimicrobial susceptibility profiles

Antimicrobial susceptibility testing showed that strain HP81 had a carbapenem-resistant multidrug-resistant phenotype (Table 1). The isolate was resistant mainly to *β*-lactams, including ertapenem and imipenem, as well as tetracycline and trimethoprim/sulfamethoxazole, but remained susceptible to several aminoglycosides, fluoroquinolones, and nitrofurantoin. Although meropenem was categorized as susceptible, its MIC was 1 μg/mL, corresponding to the upper limit of the CLSI susceptible range, whereas strain HP81 showed high MICs to ertapenem (≥8 μg/mL) and imipenem (8 μg/mL). Therefore, this phenotype should be interpreted as borderline meropenem susceptibility rather than uniformly preserved carbapenem susceptibility.

**Table 1:** Antimicrobial susceptibility of strain HP81.

Antimicrobial class	Antimicrobial agent	MIC (μg/mL)	Interpretation	S	SDD	I	R
Penicillins	Piperacillin	≥ 128	R	≤8	16	—	≥32
β-Lactam/*β*-lactamase inhibitor combinations	Amoxicillin/clavulanic acid	≥ 32	R	≤8/4	—	16/8	≥32/16
Cephalosporins	Cefazolin	≥ 64	R	≤2	—	4	≥8
	Ceftazidime	≥ 64	R	≤4	—	8	≥16
	Ceftriaxone	≥ 64	R	≤1	—	2	≥4
	Cefoperazone	16	S	≤16	—	32	≥64
Monobactams	Aztreonam	≥ 64	R	≤4	—	8	≥16
Carbapenems	Ertapenem	≥ 8	R	≤0.5	—	1	≥2
	Imipenem	8	R	≤1	—	2	≥4
	Meropenem	1	S	≤1	—	2	≥4
Aminoglycosides	Amikacin	≤ 2	S	≤4	—	8	≥16
	Gentamicin	≤ 1	S	≤2	—	4	≥8
Quinolones and fluoroquinolones	Ciprofloxacin	≤ 0.25	S	≤0.25	—	0.5	≥1
	Levofloxacin	≤ 0.25	S	≤0.5	—	1	≥2
Tetracyclines	Tetracycline	≥ 16	R	≤4	—	8	≥16
Nitrofurans	Nitrofurantoin	≤ 16	S	≤32	—	64	≥128
Folate pathway antagonists	Trimethoprim/sulfamethoxazole	≥ 320	R	≤2/38	—	—	≥4/76

S indicates susceptible; SDD, susceptible-dose dependent; I, intermediate; and R, resistant.

### 3.2 Phylogenetic position of strain HP81 within *Enterobacter soli*

To determine the phylogenetic position of strain HP81, we analyzed it together with 136 publicly available Enterobacter genomes from the NCBI database, including available *E. soli* genomes, using a core-genome phylogenetic approach. These genomes represented 77 multilocus sequence typing-defined sequence types from 25 countries and regions between 2010 and 2024 and were derived from human, animal, plant, and environmental sources. In the resulting tree, strain HP81 clustered within the *E. soli* lineage and was assigned to ST3367 (Figure 1). Among the 24 *E. soli* genomes included, only one human-derived isolate, GCF_043970025.1 from Taiwan, was identified; this isolate belonged to ST2935 and clustered near two environmental ST2935 isolates from China and Argentina. Strain HP81 formed a separate branch from these related genomes, suggesting a distinct genomic background. Given the limited number of clinical *E. soli* genomes currently available, this phylogenetic placement provides useful context for interpreting the resistance and plasmid features of strain HP81.

### 3.3 Comparative genomic features of strain HP81 among *Enterobacter soli* isolates

To compare strain HP81 with other *E. soli* isolates, we constructed a heatmap of country, year, source, and representative resistance and virulence-associated genes (Figure 2). The analyzed genomes varied in geographic origin, isolation source, and gene content, with resistance genes showing greater diversity than virulence-associated genes. Strain HP81 carried 14 resistance and virulence-associated genes, including *bla*_NDM-1_ and additional resistance genes from different antimicrobial classes, indicating a multidrug-resistant genotype. Its closest related genomes were two plant-derived isolates from the United States, GCF_051337025.1 and GCF_032054945.1; compared with these isolates, strain HP81 shared *fosA* and *oqxA* but carried additional resistance and virulence-associated genes, suggesting a more complex accessory gene profile. Strain HP81 also showed partial similarity to GCF_043970025.1, GCF_019049835.1, and GCF_052373435.1, which commonly carried *bla*OXA, *fosA*, ere(A), mph(A/R), aac(6′), *oqxA*, and sul. These shared features suggest that phylogenetically related *E. soli* isolates from human, plant, and environmental sources may have overlapping resistance-gene backgrounds, although direct transmission cannot be inferred. Overall, strain HP81 displayed a distinct multidrug-resistance profile within the currently available *E. soli* dataset.

### 3.4 Plasmid composition of strain HP81

Complete genome sequencing revealed that strain HP81 contained one chromosome and four plasmids: pHP81-1 (230,653 bp), carrying a pKPC-CAV1321-like replicon; pHP81-2 (176,882 bp), carrying an IncFIB(pENTAS01)-like replicon; pHP81-3 (54,652 bp), carrying an IncX3 replicon; and pHP81-4 (6,574 bp), carrying a Col(pHAD28)-like replicon. Among these plasmids, pHP81-3 was identified as the key carbapenem-resistance plasmid because it carried *bla*_NDM-1_. The circular maps of pHP81-1, pHP81-2, and pHP81-4 are provided in Supplementary Figure S1. This 54,652-bp IncX3 plasmid also carried *ble*, *bla*_SHV-12_, multiple insertion sequences, and transfer-associated genes, including relaxase, *virB4*, *virD4*, and pilus assembly genes (Figure 3). The other three plasmids, including the multidrug-resistance-associated pHP81-1, are summarized in Supplementary Figure S1. These findings indicate a diverse plasmid background in strain HP81 and support further analysis of pHP81-3 as the major *bla*_NDM-1_-bearing plasmid potentially involved in resistance maintenance and dissemination.

### 3.5 Comparative analysis of the *bla*_NDM-1_-bearing IncX3 plasmid pHP81-3 and related Enterobacterales plasmids

To examine the genetic background of *bla*_NDM-1_ in pHP81-3, phylogenetic analysis and comparative alignment were performed using representative related *bla*_NDM_-bearing plasmids (Figure 4). In the plasmid tree, pHP81-3 clustered with plasmids recovered from *Enterobacter hormaechei*, *Enterobacter kobei*, *Enterobacter cloacae*, and members of the *E. cloacae* complex, indicating close relatedness to *bla*_NDM_-positive plasmids from different *Enterobacter* hosts (Figure 4A). The local genetic environment of *bla*_NDM-1_ in pHP81-3 showed a mobile-element-associated resistance structure. Upstream of *bla*_NDM-1_, several mobile genetic elements were identified, including Δ*Tn3*, *IS3000*, Δ*ISAba125*, *IS30*, and *IS5*, whereas the downstream region contained the conserved gene arrangement *ble–trpF–dsbD–cutA–groES–groL*.

Comparative alignment showed that the segment *IS3000*–Δ*ISAba125*–*bla*_NDM-1_–*ble–trpF–dsbD–cutA* was highly conserved between pHP81-3 and related plasmids (Figure 4B). The *ble–trpF–dsbD–cutA* cassette was particularly stable, whereas variation was mainly observed in the outer flanking regions, including partial deletions, changes in insertion sequence number or orientation, and local rearrangements of adjacent accessory genes. These findings suggest that the *bla*_NDM-1_-associated resistance module has been maintained as a conserved genetic unit while undergoing local recombination in different plasmid backgrounds. Therefore, the observed similarity should be interpreted as plasmid relatedness and resistance-module conservation rather than direct evidence of plasmid transmission.

### 3.6 Machine-learning analysis supports the *bla*_NDM_-positive IncX3 plasmid profile of pHP81-3

**Figure 6:**
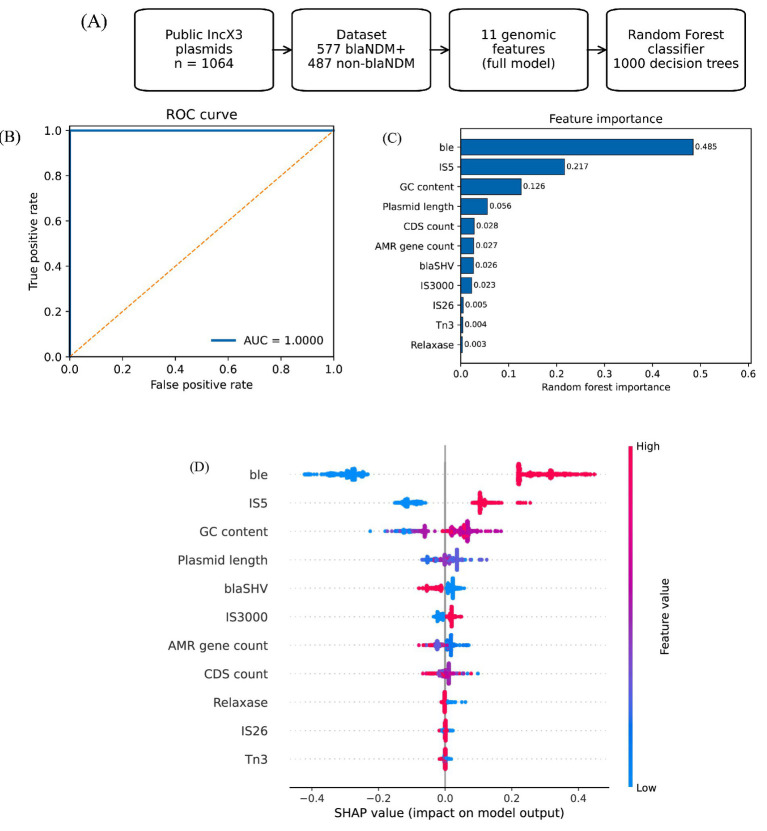
Machine-learning-assisted profiling of *bla*_NDM_-positive IncX3 plasmids. **(A)** Workflow of the analysis using 1,064 public IncX3 plasmids, including 577 *bla*_NDM_-positive and 487 non-*bla*_NDM_ plasmids. **(B)** Receiver operating characteristic curve of the full 11-feature random forest model. **(C)** Random forest feature-importance ranking. **(D)** SHapley Additive exPlanations summary plot showing feature contributions to *bla*_NDM_-positive IncX3 plasmid classification.

To quantitatively assess whether pHP81-3 conforms to a broader *bla*_NDM_-positive IncX3 plasmid profile, a machine-learning-assisted classification analysis was performed using 1,064 public IncX3 plasmids, including 577 *bla*_NDM_-positive and 487 non-*bla*_NDM_ plasmids (Figure 6A). A full 11-feature random forest model was constructed using plasmid length, GC content, coding sequence count, antimicrobial resistance gene count, *ble*, *bla*_SHV_, *IS3000*, *IS5*, *IS26*, *Tn3*, and relaxase. The model showed strong discriminatory performance, with a test-set accuracy of 0.9953, an area under the curve of 1.0000 (Figure 6B), and a five-fold cross-validation mean area under the curve of 0.9999 ± 0.0002 (mean ± SD).

Feature-importance and SHapley Additive exPlanations analyses showed that *ble*, *IS5*, GC content, plasmid length, coding sequence count, antimicrobial resistance gene count, *bla*_SHV_, and *IS3000* contributed to classification (Figures 6C,D). When the feature profile of pHP81-3 was projected into the trained classifier, the plasmid was assigned to the *bla*_NDM_-positive IncX3 plasmid profile. To reduce the possibility that this classification was dominated by the *bla*_NDM_-associated *ble* feature, a without-*ble* sensitivity model was constructed and retained high performance, with a test-set area under the curve of 0.9962 and a five-fold cross-validation area under the curve of 0.9961 ± 0.0003 (mean ± SD). Together, these results support the consistency of pHP81-3 with a conserved IncX3-*bla*_NDM_ plasmid signature.

### 3.7 Cross-species conservation of the *bla*_NDM-1_-associated resistance module

To assess the conservation of the *bla*_NDM-1_-associated region across different bacterial hosts, representative plasmids were selected from high-similarity BLASTn hits covering four Enterobacterales species: *Klebsiella pneumoniae*, *Enterobacter cloacae*, *Citrobacter freundii*, and *Escherichia coli* (Figure 5). The alignment showed that the central *bla*_NDM-1_–*ble–trpF–dsbD–cutA* region was highly conserved across plasmids from different Enterobacterales species, whereas the surrounding mobile-element-rich flanking regions showed greater variation. In pHP81-3, *bla*_NDM-1_ was embedded in an insertion sequence and transposon-associated region, and several compared plasmids retained a similar local organization. However, partial deletions, inversions, and replacement of adjacent accessory segments were observed outside the conserved core region. These findings suggest that the *bla*_NDM-1_-associated module may be maintained as a conserved genetic block across different plasmid backgrounds and bacterial hosts, while the outer flanking regions continue to evolve through local recombination.

## 4 Discussion

Carbapenem-resistant Enterobacterales are a major public health concern because carbapenemase genes are frequently disseminated by plasmids and associated mobile genetic elements (Parker et al., 2025; Shin et al., 2025). In this study, *Enterobacter soli* strain HP81 was identified as a clinical carbapenem-resistant multidrug-resistant isolate carrying four plasmids, including the *bla*_NDM-1_-bearing IncX3 plasmid pHP81-3. Clinical genomic reports of *E. soli* remain limited compared with those of other members of the *Enterobacter cloacae* complex. The identification of a *bla*_NDM-1_-bearing IncX3 plasmid in this species expands the known host background of clinically relevant carbapenem-resistance plasmids and suggests that uncommon *Enterobacter* species should not be overlooked in plasmid-resolved surveillance.

Although strain HP81 was recovered from a blood culture, the available retrospective clinical information was insufficient to establish *E. soli* as the primary cause of infection or to classify its recovery definitively as an incidental finding. Therefore, the clinical significance of this isolate remains uncertain. The resistance phenotype of strain HP81 was broadly consistent with the presence of multiple plasmid-borne *β*-lactamase and other resistance determinants, although not all detected resistance-associated genes corresponded to phenotypic resistance under the tested conditions. In addition to *bla*_NDM-1_, the strain carried several β-lactamase genes, including *bla*_SHV-12_, *bla*_OXA-1_, *bla*_DHA-1_, and *bla*_TEM-1_, as well as genes associated with resistance to aminoglycosides, quinolones, sulfonamides, tetracyclines, trimethoprim, rifampicin, chloramphenicol, and disinfectant compounds. The presence of a multidrug-resistance-associated plasmid, pHP81-1, together with the *bla*_NDM-1_-bearing plasmid pHP81-3 indicates a complex plasmid background that may contribute to resistance maintenance under antimicrobial selection (Zhong et al., 2022; Yuan et al., 2023). Therefore, the multidrug-resistant phenotype of strain HP81 is better explained by the combined contribution of multiple resistance regions rather than by *bla*_NDM-1_ alone.

The discordant carbapenem susceptibility profile of strain HP81 warrants cautious interpretation. Although meropenem was categorized as susceptible, its MIC was 1 μg/mL, corresponding to the upper limit of the CLSI susceptible range. The lower susceptible breakpoint for ertapenem (≤0.5 μg/mL) may partly contribute to the categorical discrepancy, although it does not fully explain the observed resistance to imipenem. Because NDM-1 is capable of hydrolyzing meropenem, this phenotype should not be interpreted as indicating an absence of NDM-1 activity. One plausible explanation is reduced expression of *bla*_NDM-1_. Previous studies have shown that alterations in the promoter region of *bla*_NDM-1_ can reduce NDM-1 production and consequently decrease meropenem resistance, while variation in *bla*_NDM-1_ gene or plasmid copy number may alter effective gene dosage and the resulting carbapenem-resistance phenotype (Huang et al., 2013; Cheung et al., 2021). Differences in outer membrane permeability, porin function, or efflux activity may further modify the observed phenotype. However, promoter activity, *bla*_NDM-1_ transcript or protein abundance, gene or plasmid copy number, membrane permeability, porin function, and efflux activity were not experimentally evaluated in strain HP81. Therefore, these mechanisms remain plausible explanations requiring further functional validation.

pHP81-3 was the key resistance plasmid in strain HP81 because it carried *bla*_NDM-1_ on an IncX3 backbone together with transfer-associated genes. In this plasmid, *bla*_NDM-1_ was embedded in a mobile-element-rich region, with Δ*Tn3*, *IS3000*, Δ*ISAba125*, *IS30*, and *IS5* upstream and the conserved *ble–trpF–dsbD–cutA–groES–groL* gene cluster downstream. This arrangement is consistent with common *bla*_NDM_-associated genetic environments, in which *bla*_NDM_ is linked to *ble*_MBL_ and surrounded by insertion sequences or transposon-related elements (Partridge et al., 2018; Qin et al., 2024; Alglave et al., 2025; Han et al., 2025). Comparative analysis showed that pHP81-3 was closely related to *bla*_NDM_-bearing plasmids from other *Enterobacter* species. Cross-species comparison further showed that the core *bla*_NDM-1_–*ble–trpF–dsbD–cutA* region was conserved in plasmids from *Klebsiella pneumoniae*, *Citrobacter freundii*, and *Escherichia coli*, whereas the flanking regions showed deletions, inversions, and local rearrangements. This pattern suggests that the conserved *bla*_NDM-1_-associated resistance module may be maintained across different plasmid backgrounds, while adjacent mobile-element-rich regions continue to undergo local recombination (Acman et al., 2022; Tsukada et al., 2024).

The ecological distribution of *E. soli* may also be relevant to the maintenance and dissemination of resistance plasmids. This species was originally described from a soil isolate (Manter et al., 2011), and the publicly available *E. soli* genomes analyzed in the present study included isolates from plant and environmental sources, supporting its occurrence outside clinical settings. If *E. soli* can persist in environmental or intestinal microbial communities, carriage of *bla*_NDM-1_-bearing IncX3 plasmids could allow it to act as a transient reservoir and create opportunities for plasmid exchange with more clinically prevalent Enterobacterales species, including *Escherichia coli*, *Klebsiella pneumoniae*, and members of the *Enterobacter cloacae* complex. The cross-host conservation of the *bla*_NDM-1_-associated resistance module and the presence of transfer-associated genes in pHP81-3 support the biological plausibility of this hypothesis but do not demonstrate *in situ* plasmid transfer. Because the present study did not include environmental or fecal sampling, colonization analysis, or experimental conjugation assays, the potential reservoir and donor roles of *E. soli* require further ecological surveillance and functional validation.

The machine-learning-assisted analysis provided additional quantitative support that pHP81-3 conforms to a conserved *bla*_NDM_-positive IncX3 plasmid profile. While comparative plasmid alignment demonstrated structural conservation of the *bla*_NDM-1_ genetic environment, the random forest model assessed pHP81-3 against a broader public IncX3 plasmid dataset. The full model indicated that *bla*_NDM_-positive IncX3 plasmids share recognizable genomic and mobile-element-associated features, including *ble*, *IS5*, *IS3000*, GC content, plasmid length, and other plasmid-level variables. The without-*ble* sensitivity model retained high performance after excluding the *bla*_NDM_-associated *ble* feature, suggesting that classification was not solely driven by a single resistance-linked marker. Therefore, the machine-learning results should be interpreted as profile-level support for a conserved IncX3-*bla*_NDM_ plasmid signature rather than as evidence of plasmid transmissibility or clinical spread.

This study has several limitations. First, the present study was based on a single clinical *E. soli* isolate. Therefore, the findings cannot be extrapolated to the prevalence, genetic diversity, or epidemiological distribution of *bla*_NDM-1_-carrying IncX3 plasmids in *E. soli*. Although the complete genome and comparative plasmid analyses provide a detailed genomic description of this isolate, larger multicenter studies involving clinical and environmental isolates are needed to determine whether *E. soli* represents a broader reservoir of carbapenem-resistance plasmids. Second, strain HP81 was isolated in 2019 but was retrospectively characterized in 2025 and reported in 2026. Although the strain was cryopreserved at −80 °C during this interval, the delay reduces its temporal relevance for contemporary epidemiological surveillance. Third, although *bla*_NDM-1_ was identified genomically and strain HP81 was phenotypically resistant to ertapenem and imipenem, mCIM or another specific phenotypic carbapenemase-confirmation assay was not performed. Therefore, carbapenemase production was not directly confirmed using a specific phenotypic assay. Fourth, the related plasmids used for comparative analyses were selected from public databases according to BLAST-based sequence similarity and host-representation criteria. Although this strategy enabled targeted comparisons across different Enterobacterales hosts, it was dependent on database content and selection criteria and may not capture the full diversity of related plasmids. Moreover, sequence similarity alone cannot establish direct plasmid transmission. Fifth, although pHP81-3 carried transfer-associated genes, its conjugative transferability was not experimentally confirmed. Sixth, the machine-learning model was trained using plasmid-level features derived from public genome annotations; therefore, its output reflects similarity to *bla*_NDM_-positive IncX3 plasmid profiles rather than direct evidence of horizontal transfer. Finally, incomplete source metadata for some related plasmids limited our ability to infer potential clinical–environmental transmission. Further conjugation assays, phenotypic assays for carbapenemase confirmation, plasmid stability tests, fitness-cost analyses, and broader multicenter surveillance are needed to validate and extend the present findings.

Overall, this study provides plasmid-resolved evidence that clinical *E. soli* can harbor a conserved *bla*_NDM-1_-bearing IncX3 plasmid closely related to those circulating among Enterobacterales. This finding expands the recognized host range of clinically important IncX3 resistance plasmids and highlights *E. soli* as a potentially overlooked host in carbapenem-resistance surveillance. The integration of comparative plasmid genomics with machine-learning-assisted profiling also provides a quantitative perspective for evaluating *bla*_NDM_-positive IncX3 plasmid signatures across public plasmid datasets.

## 5 Conclusion

This study identified *Enterobacter soli* strain HP81 as a clinical carbapenem-resistant multidrug-resistant isolate carrying a *bla*_NDM-1_-bearing IncX3 plasmid, pHP81-3. Plasmid-resolved comparative analysis showed that pHP81-3 was related to *bla*_NDM_-bearing plasmids from other Enterobacterales and contained a conserved *bla*_NDM-1_–*ble–trpF–dsbD–cutA* resistance module with variable flanking regions. Machine-learning-assisted profiling further supported the consistency of pHP81-3 with a conserved *bla*_NDM_-positive IncX3 plasmid signature. These findings suggest that *E. soli* may serve as an overlooked host of clinically relevant IncX3-*bla*_NDM_ resistance plasmids. Further experimental studies are needed to verify the transferability, stability, and fitness effects of pHP81-3.

## Data Availability

The genome sequencing project of Enterobacter soli strain HP81 has been registered in NCBI under BioProject accession number PRJNA1450822 and BioSample accession number SAMN57172152 (https://www.ncbi.nlm.nih.gov/bioproject/PRJNA1450822). The genome/plasmid FASTA files, machine-learning feature matrix, trained random forest models, model-performance summaries, prediction outputs, scripts, package requirements, and README file are provided as Supplementary Data S1.
