# Supplementary material for: Plasmid-resolved genomics and machine learning-assisted profiling reveal Enterobacter soli as an overlooked host of a blaNDM-1-bearing IncX3 plasmid
Source: Front Microbiol. 2026 Aug 4;17:1909491. doi: 10.3389/fmicb.2026.1909491 (PMC13481361; doi:10.3389/fmicb.2026.1909491)
Supplement: Supplementary file 1 [file Data_Sheet_2.DOCX]

Supplementary Material

# 1 Supplementary Data

**Supplementary Data 1. Machine-learning files and genome/plasmid FASTA sequences used in this study.**

This supplementary package contains the feature matrix of 1064 public IncX3 plasmids, the HP81 plasmid feature file, trained random forest models, model-performance summaries, HP81 prediction outputs, feature-importance results, SHapley Additive exPlanations output files, analysis scripts, package requirements, and a README file. The package also includes the FASTA files for the HP81 chromosome and plasmids pHP81-1, pHP81-2, pHP81-3, and pHP81-4, as well as the combined genome sequence file. These files were used to reproduce the machine-learning-assisted classification of pHP81-3 as a *bla*_NDM_-positive IncX3 plasmid profile and the without-*ble* sensitivity analysis.

# 2 Supplementary Figures

## Supplementary Figure 1. Circular maps of the additional plasmids carried by *Enterobacter soli* strain HP81.

Circular maps of the three additional plasmids carried by strain HP81, excluding the *bla*_NDM-1_-bearing IncX3 plasmid pHP81-3 shown in the main text. **(A)** pHP81-1, 230,653 bp; **(B)** pHP81-2, 176,882 bp; and **(C)** pHP81-4, 6,574 bp. Colored rings indicate nucleotide sequence similarity to representative related plasmids. Inner tracks show GC content and GC skew. Annotated features include antimicrobial resistance genes, mobile genetic elements, replication-associated genes, and transfer-associated genes where present.
