# Supplementary material for: Plasmid-resolved genomics and machine learning-assisted profiling reveal Enterobacter soli as an overlooked host of a blaNDM-1-bearing IncX3 plasmid
Source: Front Microbiol. 2026 Aug 4;17:1909491. doi: 10.3389/fmicb.2026.1909491 (PMC13481361; doi:10.3389/fmicb.2026.1909491)
Supplement: Supplementary file 2 [file Data_Sheet_1.ZIP › Supplementary Data 1/figures/full_model/RF_full_11_features_RF_feature_importance.pdf]

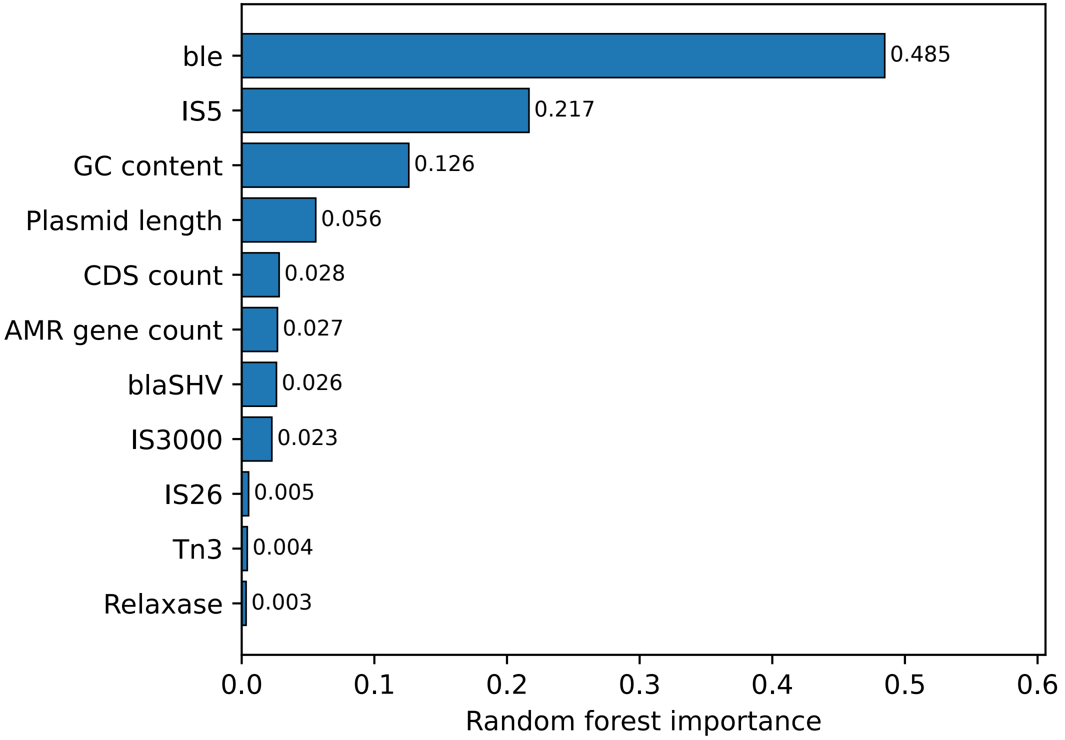
ble
IS5
GC content
Plasmid length
CDS count
AMR gene count
blaSHV
IS3000
IS26
Tn3
Relaxase
0.485
0.217
0.126
0.056
0.028
0.027
0.026
0.023
0.005
0.004
0.003
0.0
0.1
0.2
0.3
0.4
0.5
0.6
Random forest importance
