# Supplementary material for: Plasmid-resolved genomics and machine learning-assisted profiling reveal Enterobacter soli as an overlooked host of a blaNDM-1-bearing IncX3 plasmid
Source: Front Microbiol. 2026 Aug 4;17:1909491. doi: 10.3389/fmicb.2026.1909491 (PMC13481361; doi:10.3389/fmicb.2026.1909491)
Supplement: Supplementary file 2 [file Data_Sheet_1.ZIP › Supplementary Data 1/figures/full_model/RF_full_11_features_SHAP_bar.pdf]

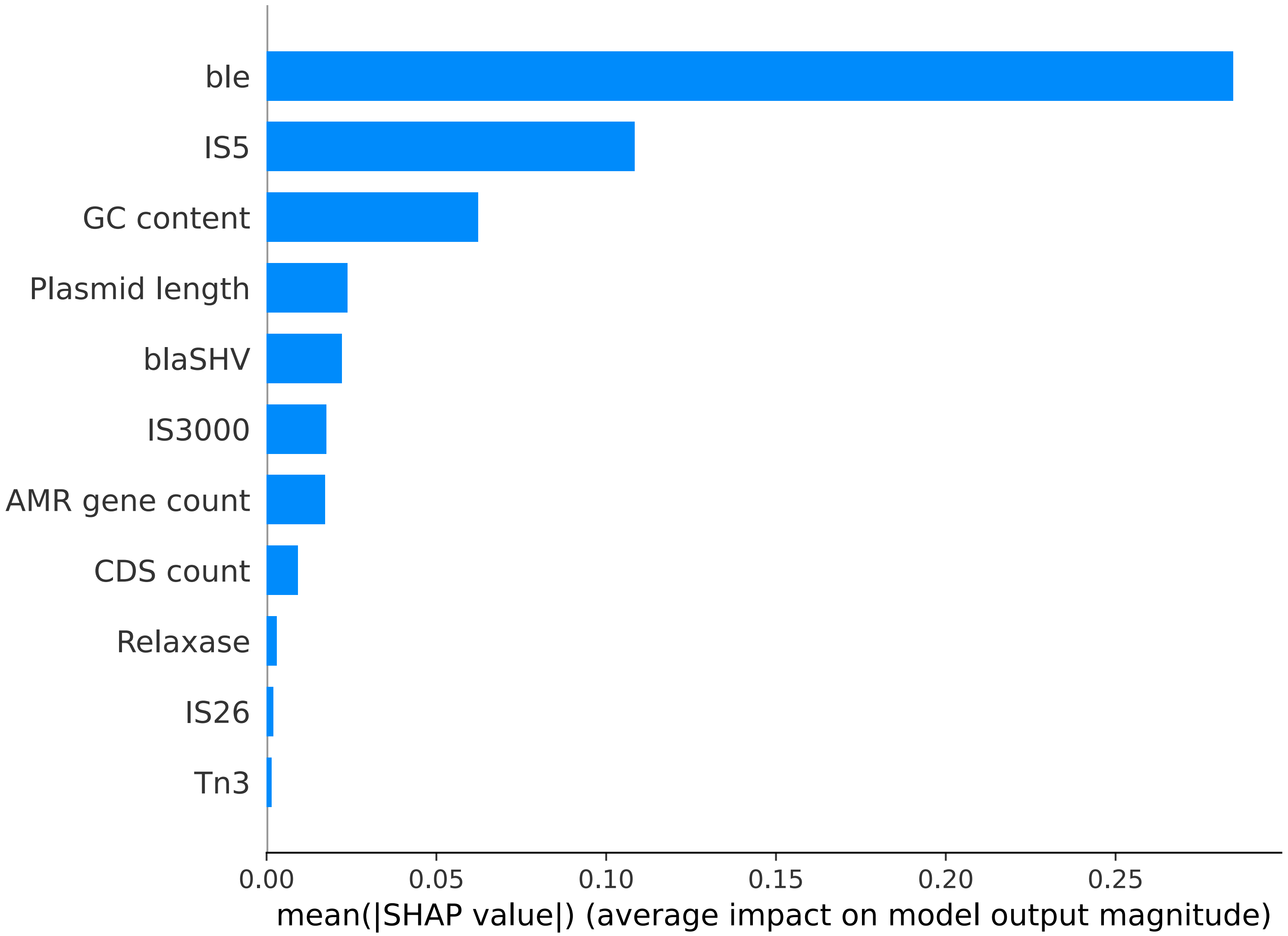

ble
IS5
GC content
Plasmid length
blaSHV
IS3000
AMR gene count
CDS count
Relaxase
IS26
Tn3
0.00
0.05
0.10
0.15
0.20
0.25
mean(|SHAP value|) (average impact on model output magnitude)
