# Supplementary material for: Plasmid-resolved genomics and machine learning-assisted profiling reveal Enterobacter soli as an overlooked host of a blaNDM-1-bearing IncX3 plasmid
Source: Front Microbiol. 2026 Aug 4;17:1909491. doi: 10.3389/fmicb.2026.1909491 (PMC13481361; doi:10.3389/fmicb.2026.1909491)
Supplement: Supplementary file 2 [file Data_Sheet_1.ZIP › Supplementary Data 1/figures/full_model/RF_full_11_features_workflow.pdf]

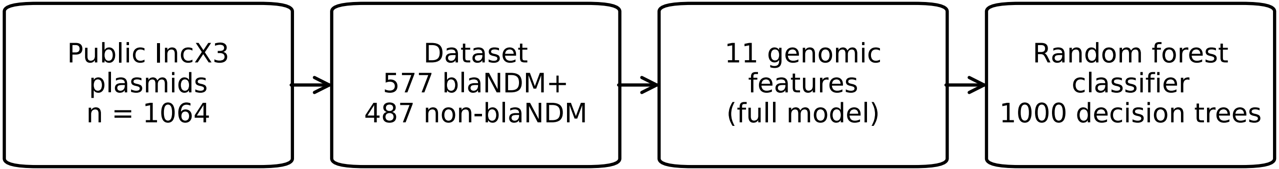
Public IncX3
plasmids
n = 1064
Dataset
577 blaNDM+
487 non-blaNDM
11 genomic
features
(full model)
Random forest
classifier
1000 decision trees
